# Coherent Spin and Quasiparticle Dynamics in Solution‐Processed Layered 2D Lead Halide Perovskites

**DOI:** 10.1002/advs.201800664

**Published:** 2018-08-13

**Authors:** David Giovanni, Wee Kiang Chong, Yu Yang Fredrik Liu, Herlina Arianita Dewi, Tingting Yin, Yulia Lekina, Ze Xiang Shen, Nripan Mathews, Chee Kwan Gan, Tze Chien Sum

**Affiliations:** ^1^ Energy Research Institute @ NTU ERI@N Interdisciplinary Graduate School Nanyang Technological University 50 Nanyang Avenue, Block S2‐B3a‐01 Singapore 639798 Singapore; ^2^ Division of Physics and Applied Physics School of Physical and Mathematical Sciences Nanyang Technological University 21 Nanyang Link Singapore 637371; ^3^ Institute of High Performance Computing 1 Fusionopolis Way, #16‐16 Connexis Singapore 138632; ^4^ Theory of Condensed Matter Group Cavendish Laboratory JJ Thomson Avenue Cambridge CB3 0HE UK; ^5^ Energy Research Institute @NTU (ERI@N) Research Techno Plaza, X‐Frontier Block, Level 5, 50 Nanyang Drive Singapore 637553; ^6^ School of Materials Science and Engineering Nanyang Technological University Nanyang Avenue Singapore 639798

**Keywords:** 2D perovskites, coherent phonon dynamics, layered perovskites, pump‐probe spectroscopy, transient absorption

## Abstract

Layered 2D halide perovskites with their alternating organic and inorganic atomic layers that form a self‐assembled quantum well system are analogues of the purely inorganic 2D transition metal dichalcogenides. Within their periodic structures lie a hotbed of photophysical phenomena such as dielectric confinement effect, optical Stark effect, strong exciton–photon coupling, etc. Detailed understanding into the strong light–matter interactions in these hybrid organic–inorganic semiconductor systems remains modest. Herein, the intricate coherent interplay of exciton, spin, and phonon dynamics in (C_6_H_5_C_2_H_4_NH_3_)_2_PbI_4_ thin films using transient optical spectroscopy is explicated. New insights into the hotly debated origins of transient spectral features, relaxation pathways, ultrafast spin relaxation via exchange interaction, and strong coherent exciton–phonon coupling are revealed from the detailed phenomenological modeling. Importantly, this work unravels the complex interplay of spin–quasiparticle interactions in these layered 2D halide perovskites with large spin–orbit coupling.

Halide perovskites are in the limelight due to their outstanding optoelectronic properties for photovoltaics^1^ and light emitting diodes (LEDs).^2^, ^3^, ^4^ Layered 2D halide perovskites, comprising of alternating organic and inorganic layers, is one subcategory within this family of hybrid materials. Specifically, for (RNH_3_)_2_MX_4_, where RNH_3_^+^ is an organic cation such as C_6_H_5_C_2_H_4_NH_3_^+^; M is a divalent metal such as Pb^2+^ or Sn^2+^; and X is a halide such as Br^−^ and I^−^, the [MX_6_]^4−^ octahedron layer is sandwiched between two organic cation RNH_3_^+^ layers, yielding a sheet with a “quantum well” (QW)‐like configuration. Successive sheets bind together by van der Waals interaction, which can be exfoliated to form single or few layers.^5^, ^6^ These 2D perovskites share many similarities with transition metal dichalcogenide monolayers and can be considered an analogue.

Presently, most studies in halide perovskites focused on the (RNH_3_)MX_3_ 3D perovskites. However, there are growing interests in 2D perovskites due to their ambient moisture resilience over the less stable 3D perovskites.^7^, ^8^, ^9^ Such natural type‐I QW structures combined with their chemical versatility and facile tunability are highly desirable for optoelectronic applications. Specifically, a new family of quasi‐2D Ruddlesden–Popper (RP) perovskites have now shown to exhibit favorable photovoltaic efficiency^7^, ^8^ and LED performance^9^ with excellent moisture stability. Herein, we study the photophysics of the most basic configuration of RP perovskite family, i.e., the pure 2D perovskite phase or *n* = 1, which is the building block of RP perovskites.

Apart from these classic applications, 2D perovskites with their exotic structures, large exciton binding energies and strong spin–orbit coupling (due to Pb) also afford exciting possibilities to study the rich photophysics such as dielectric confinement effect,^10^, ^11^ optical Stark effect,^12^ strong exciton–photon coupling,^13^, ^14^ etc. Early photophysics studies in 2D perovskites were mainly limited to their linear properties;^10^, ^15^, ^16^ while studies on their transient photophysics are few and far between.^17^, ^18^, ^19^ In particular, transient optical spectroscopy takes on a leading role in understanding these unusual phenomena and in unlocking the latent potential of these novel materials.^20^ For instance, their potential for spin‐based applications was first uncovered by transient photophysics studies,^21^ and latter affirmed by theoretical calculations.^22^, ^23^ A clear understanding of the carrier and quasiparticle dynamics in these exotic systems is key to realizing their full potential for new disruptive optoelectronic technologies.

In this study, we explicate the origins of the transient spectral features, coherent interactions, and dynamics between the photoexcited excitons, their spin and phonon subsystems in solution‐processed (C_6_H_5_C_2_H_4_NH_3_)_2_PbI_4_ perovskite (hereafter termed PEPI) thin films using femtosecond (fs) transient absorption (TA) spectroscopy. Our findings reveal an ultrafast exciton spin relaxation via exchange Coulomb interaction. Surprisingly, strong coherent phonon oscillation with a frequency of 1.15 ± 0.05 THz (from the exciton–longitudinal‐optical (LO) phonon interaction) are also observed in these solution‐processed polycrystalline thin films, which is typically seen only in single crystals^24^, ^25^ or crystalline nanostructures.^26^ This phonon mode correlates well with Raman spectroscopy and first‐principles lattice dynamics calculations. Furthermore, we developed a phenomenological model which could satisfactorily replicate the experimental transient dynamics in PEPI. Importantly, our work provides the first in‐depth understanding into the coherent transient exciton dynamics and interplay of spin–quasiparticle interactions in this novel layered 2D perovskites.

**Figure** **1**a shows the schematic crystal structure of PEPI. The linear absorption spectrum of PEPI is provided in the Figure 1b top, showing a strong excitonic feature at 517 nm. Figure 1b bottom shows the room temperature broadband transient absorption spectra for a PEPI thin film excited using above bandgap 400 nm pump with fluence of 8 µJ cm^−2^ at *t* = 5 ps. A strong excitonic photobleaching (PB, Δ*T*/*T* > 0) peak was observed at ≈517 nm. In addition, two photoinduced absorption (PA, Δ*T*/*T* < 0) peaks at ≈505 and ≈525 nm, labelled as PA1 and PA2, respectively, were also observed. Almost identical transient features were also observed by resonant excitation (i.e., 515 nm pump—Figure S3a, Supporting Information). Since resonant pump would excite only (or predominantly) excitons due to momentum and energy conservation, it implies that the observed features mainly originated from the excitonic state—Section 2, Supporting Information. Insignificant difference between the rise‐time of excitonic bleaching due to 400 and 515 nm pump also implies ultrafast hot carrier cooling and exciton formation (<50 fs), which is beyond our temporal resolution (Figure S3b, Supporting Information).

**Figure 1:**
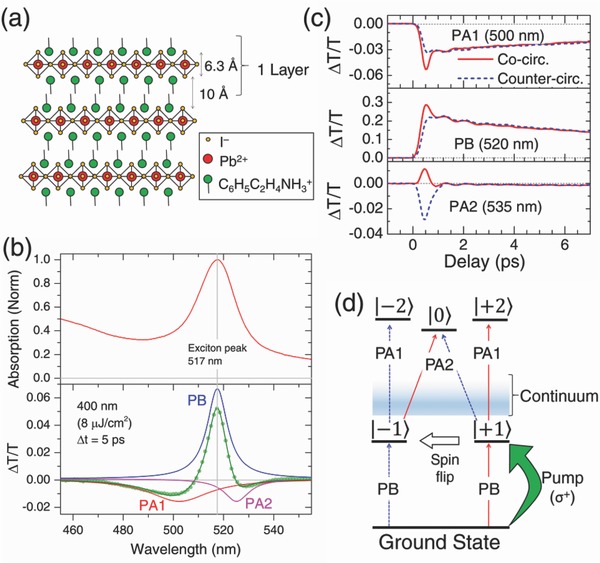
Transient Absorption spectral features in PEPI. a) Schematic structure of PEPI. b) (top) Linear absorption of PEPI and (bottom) TA spectrum at *t* = 5 ps pumped by 400 nm (8 µJ cm^−2^) and fitted with three Lorentzian peaks. Both spectra feature excitonic resonance at 517 nm. c) Kinetics at 500, 520, and 535 nm pumped by 515 nm (21 µJ cm^−2^) with co‐ (solid red) and counter‐ (dashed blue) circularly polarized pump‐probe. d) Transient optical transition model, with the *m_J_* of the states labeled.

Further analysis reveals that PB, PA1, and PA2 are well‐fitted with three Lorentzian peaks, suggesting the excitonic origins of the peaks. Global fitting of the fluence dependent TA data yielded kinetics with similar lifetimes and ratios of their decay amplitudes (see Figure S4, Supporting Information). This strongly suggests that they originate from a similar exciton state at 2.40 eV. The PB peak, which matches the excitonic peak in the linear absorption spectrum (Figure 1b), can be straight‐forwardly assigned to originate from the exciton bleach, given the dominant presence of excitons in PEPI (i.e., large exciton binding energy ≈180 meV).^27^ On the other hand, the origins of the two PA peaks on both sides of the PB peak are less clear. Observations of PA beside the PB signature in other excitonic systems have been previously reported and assigned to carrier‐induced red‐shift or peak broadening,^28^ which resulted in similar “wing‐like” PA bands (Figure S1, Supporting Information). Nevertheless, the relatively low fluence used in our experiments and the invariance of the FWHM and peak positions of our peaks dispute such assignments. The detailed discussion of the assignment is presented in Section 1 of the Supporting Information.

Meanwhile, for excitonic perovskites systems, similar PA features were also reported, in which PA2 was controversially assigned to either biexciton induced level red‐shifting,^29^ interplay of subsequent red‐ and blue‐shifting due to bandgap renormalization and hot‐carrier cooling,^30^ or even hot‐carrier induced Stark effect.^31^ These PA signatures are also distinct from the optical stark effect induced shift reported in our earlier work,^12^ in which below bandgap off‐resonant photoexcitation was used. As of now, the origins of these PA bands in perovskites remain highly controversial, especially their fast sub‐ps component (i.e., PA2). This work by white light TA measurements with resonant excitation and circular polarization control in PEPI would help clarify the origins and mechanisms of these PA bands in excitonic perovskites.

The exciton state of PEPI, with total magnetic quantum number of *m_J_* = ±1, is doubly degenerate due to the spin degree of freedom.^12^, ^32^ Using circularly polarized light (i.e., Δ*m_J_* = ± 1), we selectively pump and probe the desired exciton *J*‐(spin) states. Figure 1c shows the TA kinetics of resonantly pumped co‐ and countercircular pump‐probe signal excited by 515 nm (21 µJ cm^−2^) at these peaks. At early times (*t* < 1 ps), there are distinct differences between the polarizations, originating from the spin‐selective excitation. The cocircular PA1 (500 nm) and PB (520 nm) signals exhibit initially stronger negative and positive signal amplitudes, respectively (i.e., solid red line), as compared to their countercircular signals. Meanwhile cocircular PA2 (535 nm) exhibits an initially positive signal (i.e., solid red line), while countercircular PA2 display a much stronger negative signal amplitude (i.e., blue dashed line). This initially positive cocircular signal at 535 nm could be due to spectral overlap between the tail of initially‐stronger positive cocircular PB peak (i.e., stimulated emission or bleaching signal from the tail states), with the initially weaker negative cocircular PA2 transition.

Herein, if PA2 originated from any of the previous assignments,^29^, ^30^, ^31^ one would expect an “in‐phase” initially stronger cocircular PA2 signal, similar to PA1 and PB; because those assignments considered only one optical transition: from the ground state to an evolving exciton state (i.e., red‐/blue‐shifting, broadening, etc.—see Section 1, Supporting Information). Nevertheless, our observation of “reversed” behavior of PA2 to PA1 and PB therefore ruled out those assignments. For both co‐ and countercircular polarizations, the kinetics then merge within 1 ps, which implies ultrafast (sub‐ps) spin relaxation. Note that the observed oscillatory signal originates from the strong exciton–phonon coupling, which will be discussed later. No spin polarization dependent signature is observed using circularly polarized 400 nm excitation.

These transient signatures can be explained using our proposed model for excitonic states with their *m_J_* states as shown in Figure 1d. Without any loss of generality, we assume photoexcitation by σ^+^ pump populates only the | + 1〉 state. The populated | + 1〉 yields an initially stronger positive signal of σ^+^ PB probe due to state‐filling. This rise is quickly followed by a concomitant decay for σ^+^ PB signal and a rise of σ^−^ PB probe, which later merge together due to exciton spin relaxation. Similar behavior is observed in PA1. Since the transition originate from the exciton state at 2.40 eV, conservation of angular momentum will only allow excited‐state absorption of σ^+^ probe with Δ*m_J_* = +1. This implies that the initially‐stronger PA1 σ^+^ (cocircular) probe signal originates from | + 1〉 to | + 2〉 transitions. Spin relaxation to | − 1〉 then allows the | − 1〉 to | − 2〉 transition to occur, which is detected by σ^−^ (countercircular) probe. In the case of PA2, the initially stronger negative signal of σ^−^ (countercircular) probe originates from | + 1〉 to |0〉 transition (i.e., Δ*m_J_* = −1). Spin relaxation to | − 1〉 then allows | − 1〉 to |0〉 transition to occur, which then detected as σ^+^ (cocircular) negative signal. These upper states are optically inaccessible from the ground state via linear absorption (i.e., Δ*m_J_* = 0, ± 2).

**Figure** **2**a shows the global fit of the fluence dependent kinetics at 517 nm following 400 nm pumping, which yielded four representative lifetimes: τ_1_ = 0.25 ± 0.01 ps; τ_2_ = 10 ± 3 ps; τ_3_ = 110 ± 20 ps; and τ_4_ = 2000 ± 600 ps. The contribution of each lifetime component is plotted in Figure 2b. Herein, τ_1_ is assigned to the exciton thermalization process, which is density independent (since *A*_1_ remains nearly invariant with fluence). τ_2_ and τ_3_, which are assigned to the exciton–exciton annihilation and monomolecular (geminate) recombination processes, respectively, are density dependent and exhibits a concomitant behavior [indicated by the weight transfer from *A*_3_ to *A*_2_] with increasing fluence. Lastly, τ_4_ originates from a trapping/detrapping process. All relaxation components are simultaneously fitted with a phenomenological model which describes all four processes (see —Sections 4 and 5 of the Supporting Information for the details on the assignment and model). The model shows an excellent agreement, within 1σ confidence level (shaded area) with the experimental data. Our fitting yields trap density of (2.1 ± 0.3) × 10^17^ cm^−3^, comparable to typical 3D perovskites thin films.^20^, ^33^ Moreover, a small population of dark excitons (*m_J_* = 0, ≈2%) is also elucidated by the model. This result highlights the contributions of trap states and dark states in exciton dynamics of 2D perovskite system.

**Figure 2:**
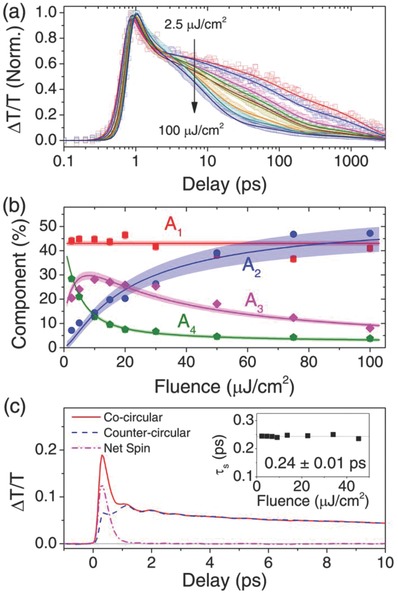
Exciton and spin dynamics in PEPI. a) Fluence dependent TA kinetics following 400 nm excitation and 515 nm probe. b) Contribution of each lifetime component versus pump fluence: *A*_1_ (red square), *A*_2_ (blue circle), *A*_3_ (magenta diamond), and *A*_4_ (green pentagon). All components are fitted simultaneously with phenomenological model (see the Supporting Information). The shaded area signifies the 1σ confidence of the fitting. c) TA kinetics resonantly pumped (9.1 µJ cm^−2^) and probed by 517 nm. The co‐ and countercircular pump‐probe polarization together with their difference, plotted in solid red, dashed blue, and dashed‐dot magenta lines, respectively. Inset: exciton spin lifetime τ_s_ as a function of pump fluence.

Figure 2c shows the resonantly pumped TA kinetics and probed at 515 nm, which yields the photoexcited exciton spin dynamics. Inset shows the net spin lifetime τ_s_ of 0.24 ± 0.01 ps (fluence independent). There are two established exciton spin relaxation processes: (i) direct simultaneous (monomolecular) electron and hole spin flip due to exchange interaction; and (ii) indirect (bimolecular) sequential spin flip of single particle.^34^ A huge longitudinal‐transverse splitting of 50 meV (i.e., 500× of GaAs) has been reported in PEPI.^11^ which suggest a strong exchange Coulomb interaction. Likewise, resonant excitation only generates monomolecular excitons, hence this will also exclude process (ii). Moreover, the individual electron (hole) spin lifetime of 3D CH_3_NH_3_PbI_3_ perovskites is ≈4 ps,^21^ which is >10× longer than exciton spin lifetime observed here. We therefore attribute of the exciton spin relaxation mechanism to (i) spin‐flip by exchange Coulomb interaction.^34^, ^35^

Lastly, we focus on the oscillations observed in the TA kinetics. **Figure** **3**a top shows the first 10 ps of PEPI thin film photoexcited at 400 nm (8 µJ cm^−2^) at five different probe wavelengths. The oscillatory signals are strongest at the crossing points of PA1/PB (508 nm) and PB/PA2 (523 nm) (see Figure 1b). Away from the crossing points, the oscillatory signals diminish. The fittings of the oscillatory component yields oscillation frequency of 1.15 ± 0.05 THz (corresponds to energy of 4.8 ± 0.4 meV) and decay lifetime of 1.2 ± 0.2 ps, independent of excitation fluence. We posit that the oscillations arise from the coherent interactions between the excitons and the phonons.

**Figure 3:**
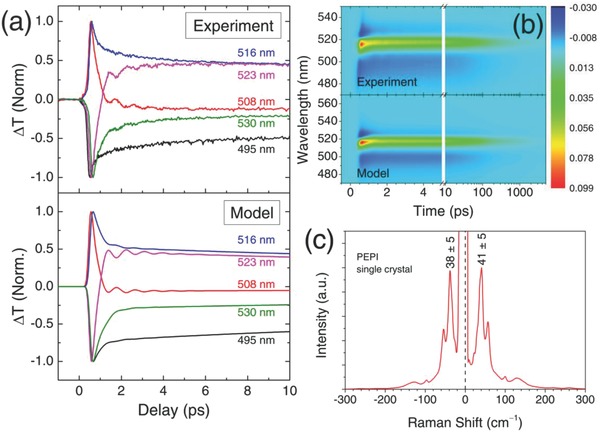
Modeling of transient features and dynamics in PEPI. a) The experimental result (top) and model (bottom) of the first 10 ps kinetics at five different probe wavelengths. b) The experimental result and model of Δ*T*/*T* of TA spectrum of pumped by 400 nm. c) Low energy Raman study on PEPI single crystal, showing a labeled pair of Stokes and anti‐Stokes with energies matches to the oscillation frequency.

The observations of strong oscillations near the crossing points can only be accounted by the small oscillations of the exciton energy (≈2 meV) due to coherent exciton–phonon coupling. To validate our assignment, we propose the following phenomenological model to test our experimental data

(1) $$\frac{\Delta T}{T}=\sum_{i=1}^{3}L_{i}\left(\lambda ,t\right)\sum_{j=1}^{4}\frac{C_{ij}}{2}H_{j}\left(t\right)e^{-t/\tau_{j}}$$

Here, *L_i_*(λ,*t*) is the Lorentzian function, which corresponds to each of the optical transition (i.e., PA1, PB, and PA2); *H_j_* (*t*) = [1 + erf(*t*/*r* − *r*/2τ_*j*_)]/2  is the rising function; *C_ij_* is constant and τ_*j*_ is the dynamic lifetime. The oscillatory component is embedded into *L_i_*(λ,*t*). The details of the model can be found in Section 6 of the Supporting Information. The result (Figure 3a bottom) yields a signal with notable oscillations only at the crossing points (i.e., at 508 and 523 nm). Figure 3b shows the Δ*T*/*T* spectrum based on our model in Equation (1), with good agreement (*R* ^2^ = 0.973) between the model and experimental data. It is noteworthy that similar but much stronger oscillatory behavior is also observed for resonant pumping (Figure 2c). In this case, the oscillations can still be observed at probe wavelengths away from the crossing points, but with similar frequencies and decay lifetimes.

Further insights were gained from low energy Raman measurements on single‐crystal PEPI samples, as our spin‐coated thin‐film samples were too thin to obtain appreciable Raman signal. The result is shown in Figure 3c, where a pair of Stokes (41 ± 5 cm^−1^ = 5.1 ± 0.6 meV) and anti‐Stokes peaks (38 ± 5 cm^−1^ = 4.7 ± 0.6 meV) are observed. Identical Raman features for wavenumber ν < 100 cm^−1^ are also obtained from the dropcasted sample, which implies that they originate from intrinsic vibrational modes and are morphology‐independent (Section 8, Supporting Information). The energy of these peaks corresponds very well with the energy of the TA oscillatory component, implying that it is related to the vibrational/phonon states. Ultrafast pump excitation induces formation of coherent phonons, which interact strongly with the excitons. First‐principles phonon calculations by Vienna Ab initio Simulation Package on PEPI structures (Section 7, Supporting Information) verify the *A*_g_ and *B*_g_ phonon modes at 39.2 and 38.7 cm^−1^, respectively—consistent with our Raman and TA results. Both modes originate from the twisting of the organic cations. This interesting finding shows that the coherent vibrations in the organic layers could influence the excitons in the inorganic layers. We further speculate that the coherent distortion of the lattice by phonons changes the eigenenergies of the excitons, resulting that in exciton energy oscillations via Raman tensor.^36^ However, we do not rule out the possibility of other mechanisms, e.g., piezoelectric modulation.^37^ But this is beyond the scope of this work.

In summary, our work explicates the room temperature ultrafast transient dynamics involving coherent interplay of excitons, spins and phonons in PEPI thin films. We elucidated the observed transient optical transitions; nonequilibrium exciton relaxation pathways; ultrafast spin relaxation via exchange Coulomb interaction; and strong coherent exciton–phonon coupling which manifests in the oscillatory behaviour of the exciton energy level. Several future research directions could be derived from our findings. First, the role of dark excitons in 2D perovskites for PV applications. Dark states might hold the key to improve charge extraction efficiency in the context of solar cell.^38^ While the presence of dark states in neat PEPI is not significant (≈2%), further systematic studies on the sample engineering (e.g., additive, solvent treatment, etc.) could be done to tune the dark states contribution, for instance, in the quasi 2D–3D Ruddlesden–Popper systems. Second, it is the role of exciton–exciton annihilation and trapping for 2D perovskite LED applications, which are the significant loss channels that impede efficient light emission. Further sample engineering could also be done to reduce these loss channels and improve its efficiency. Lastly, it is the possibility for novel optospintronics applications with 2D perovskites with magnetic doping. Photoexcited spin‐polarized excitons in perovskites might cause reordering of dopant's magnetization, from which optically controlled “spin‐torque” switches could be constructed. Such device would find applications in future technologies such as quantum computing, nonvolatile memory devices, etc. Our results hence establish the fundamental understanding of the transient photophysics in layered 2D perovskites, which is essential to explore their untapped potential.

## Experimental Section

*Sample Preparation*: The solution‐processed PEPI thin films of thickness ≈50 ± 5 nm (i.e., 25–35 alternating layers) were fabricated with stoichiometric ratios of C_6_H_5_C_2_H_4_NH_3_I and PbI_2_ precursors in *N*,*N*‐Dimethylformamide (12.5 wt% concentration). The samples were spin‐coated (4000 rpm for 30 s) on cleaned quartz substrates and annealed at 100 °C for 30 min. The samples were kept in a nitrogen‐filled environment at all times. The single‐crystal PEPI sample was prepared by first slowly mixing stoichiometric amount of the amine with hydrochloric iodide in ethanol at 0 °C, followed by stirring the solution for an hour. The solvents were evaporated, and the solid was washed with diethyl ether and dried to form C_6_H_5_C_2_H_4_NH_3_I. To obtain crystalline PEPI, stoichiometric amounts PbI_2_ and the prepared C_6_H_5_C_2_H_4_NH_3_I were premixed and dissolved in γ‐butyrolactone. The solution was filtered through PTFE filter (0.2 µm) to remove any insoluble particles. The vial was then transferred into a larger vial containing dichloromethane. Slow solvent evaporation over one week to the precursor solution gave orange crystals. All reagents were obtained from Sigma‐Aldrich.

*Transient Absorption Spectroscopy (TAS)*: Room‐temperature fs‐TAS was performed using a commercial pump‐probe setup (*Ultrafast System HELIOS*) driven by *Coherent LEGEND* laser system with repetition rate of 1 kHz, fundamental wavelength 800 nm and pulse width of ≈100 fs. The tunable pump beam was from an optical parametric amplifier; while the white light probe beam was generated from a sapphire crystal, with a 750 nm short‐pass filter used to block the 800 nm residual.

*Raman Spectroscopy*: Low wavenumber Raman measurements were carried out on a WITec CRM200 confocal microscope system at room temperature with a linearly polarized 633 nm excitation source. An Olympus objective lens (100×, NA = 0.95) was used. The laser power was set as 30 µW. The signal integration time was set to be 5 s.

## Conflict of Interest

The authors declare no conflict of interest.

## Supporting information

Supplementary
