# Supplementary material for: Coherent Spin and Quasiparticle Dynamics in Solution‐Processed Layered 2D Lead Halide Perovskites
Source: Adv Sci (Weinh). 2018 Aug 13;5(10):1800664. doi: 10.1002/advs.201800664 (PMC6193146; doi:10.1002/advs.201800664)
Supplement: Supplementary file 1 — Supplementary [file ADVS-5-1800664-s001.pdf]

Copyright WILEY-VCH Verlag GmbH & Co. KGaA, 69469 Weinheim, Germany, 2018.

ADVANCED SCIENCE
Open Access

Supporting Information

for *Adv. Sci.,* DOI: 10.1002/advs.201800664

Coherent Spin and Quasiparticle Dynamics in Solution-Processed Layered 2D Lead Halide Perovskites

*David Giovanni, Wee Kiang Chong, Yu Yang Fredrik Liu, Herlina Arianita Dewi, Tingting Yin, Yulia Lekina, Ze Xiang Shen, Nripan Mathews, Chee Kwan Gan, and Tze Chien Sum**

Supporting Information

Coherent spin and quasi-particle dynamics in solution-processed layered 2D lead halide perovskites

David Giovanni[1,2], *Wee Kiang Chong*[1,2], *Yu Yang Fredrik Liu*[3,4], *Herlina Arianita Dewi*[5], *Tingting Yin*[2], *Yulia Lekina*[2], *Ze Xiang Shen*[1,2], *Nripan Mathews*[5,6], *Chee Kwan Gan*[3] *and Tze Chien Sum*[1,2]*

[1]Energy Research Institute @ NTU, ERI@N, Interdisciplinary Graduate School, Nanyang Technological University, Singapore

[2]Division of Physics and Applied Physics, School of Physical and Mathematical Sciences, Nanyang Technological University, 21 Nanyang Link, Singapore 637371

[3]Institute of High Performance Computing, 1 Fusionopolis Way, #16-16 Connexis, Singapore 138632

[4]Theory of Condensed Matter Group, Cavendish Laboratory, JJ Thomson Avenue, Cambridge, United Kingdom CB3 0HE

[5]Energy Research Institute @NTU (ERI@N), Research Techno Plaza, X-Frontier Block, Level 5, 50 Nanyang Drive, Singapore 637553

[6]School of Materials Science and Engineering, Nanyang Technological University, Nanyang Avenue, Singapore 639798

*Corresponding Author: T.C.S.: Tzechien@ntu.edu.sg

1. Spectral signatures of various processes

Table S1 shows the known possible transient processes that can occur in an excitonic system, and their expected spectral signatures which is observed in TAS. These signal signatures are obtained by subtracting the absorbance of the sample when the pump is on with when the pump is off (*i.e.* $\Delta A = A_{on} - A_{\text{off}}$). The $\Delta T/T$ is related to ΔA by $\Delta T/T = 10^{-\Delta A} - 1$. Excitonic bleaching is modelled by Lorentzian peak.

Table S1. Expected transient signatures which would be observed by TA of various processes. In the third column, the dashed line is the sample absorbance without pump, while solid red line is the absorption with pump. The fourth column shows the transient absorption with x-axis as the probe wavelength and the y-axis as the $\Delta T/T$ signal.

Process	Possible Cause(s)	Absorption Before/After	Transient Absorption
Bleaching	State-filling; stimulated emission.	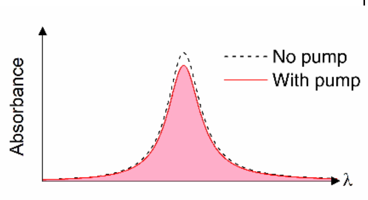	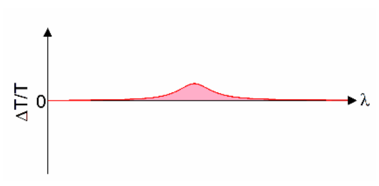
Photoinduced absorption	Excited state absorption.	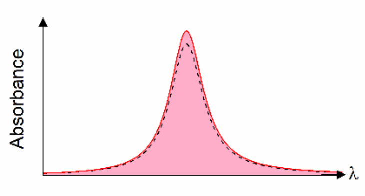	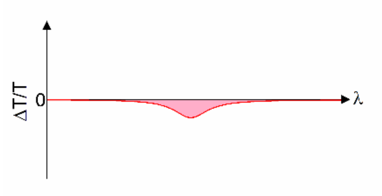
Broadening	Carrier-induced broadening.[1]	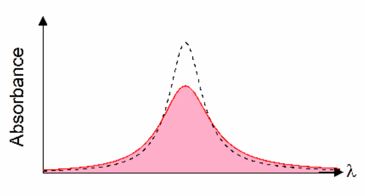	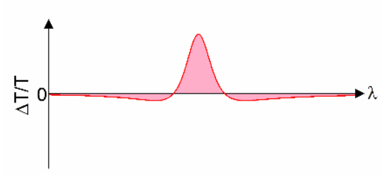

Blue-shift	Optical Stark effect;[2] hot-carrier cooling.[3]	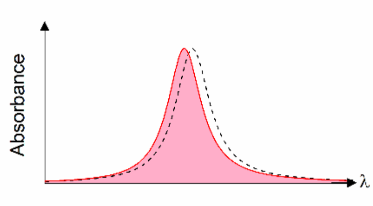	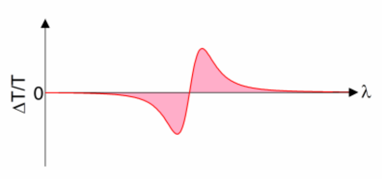
Red-shift	Bandgap renormalization;[3] bi-exciton-induced red-shifting;[4] hot-carrier-induced Stark effect.[5]	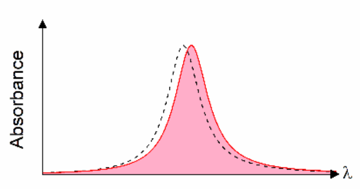	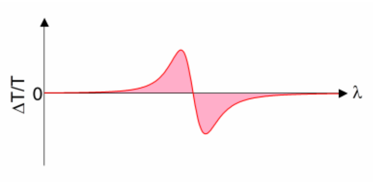

For our case of PEPI thin film, the transient spectrum is assigned to three different transient optical transitions (PA1, PB, and PA2) as modelled in Fig. 1(d) in the Main Text. These three transitions can be fitted with three Lorentzian peaks. There are four reasons why we would assigned them to three Lorentzian peaks originating from three different transitions, and not to broadening or carrier-induced redshift/blueshift:

i. The constant FWHM and peak positions of the three fitted Lorentzian peaks. Since the observed transient signal is exciton-density-dependent, the effect should diminish with time as the exciton population is depleted due to recombination. Therefore, if broadening, blue-shift or redshift were the case, there should be a decrease of the transient peaks' FWHMs (for the case of broadening) or a shifting of the transient peak positions (for the case of Blueshift or Redshift) with the decreasing transient exciton density in time. However, no of such effect were observed in the experiment.

ii. If it was a Blueshift or a Redshift process, the transient signal would have been well-fitted by two identical Lorentzian peaks with different signs at different peak positions

[*i.e.*, ΔT ∝ initial absorption + negative shifted absorption after pump excitation, revised Fig. S1(a) and (b)]. Similarly, PB/PA + broadening effect from a single optical transition would also have been well-fitted with two Lorentzian peaks with different signs at similar peak positions [Fig. S1(c)]. Even a combined PB/PA + Redshift/Blueshift + Broadening processes from a single optical transition would also have been well-fitted by two Lorentzian peaks with different signs [Fig. S1(d) to (f)]. **Since our data could only be described by three Lorentzian peaks** (and all at different peak positions), at least two different optical transitions are involved.

iii. Difference in the early time dynamics of the first three peaks. If the three transient features come from a single broadened or/and shifted optical transition, they would have shown similar polarization dependent dynamics. The fact that PA2 shows opposite behaviour from PA1 and PB peaks, means that at least PA2 comes from different optical transition as PA1 and PB, as assigned in the manuscript.

iv. The peak positions of the three features are too far apart to come from exciton/laser induced shifting. PA1 and PB peaks are 73 meV apart, while PB and PA2 peaks are 36 meV apart. The typical exciton population induced blue/redshift is expected to be only < 10 meV. Assigning such process to level Blueshift/Redshift would be unphysical.

Based on the reasons (ii) and (iii), there are only two possible cases which could explain the transient spectrum: case (a) 2 PA + 1 PB peaks, as described in the manuscript; or case (b) PB + blueshift + broadening of the ground state to exciton state transition (which account for PA1 and PB features) + PA2 peak from exciton state to the upper state transition. However, because of the reasons (i) and (iv), hence case (b) can be eliminated. Hence, we conclude that case (a) is the most plausible assignment.

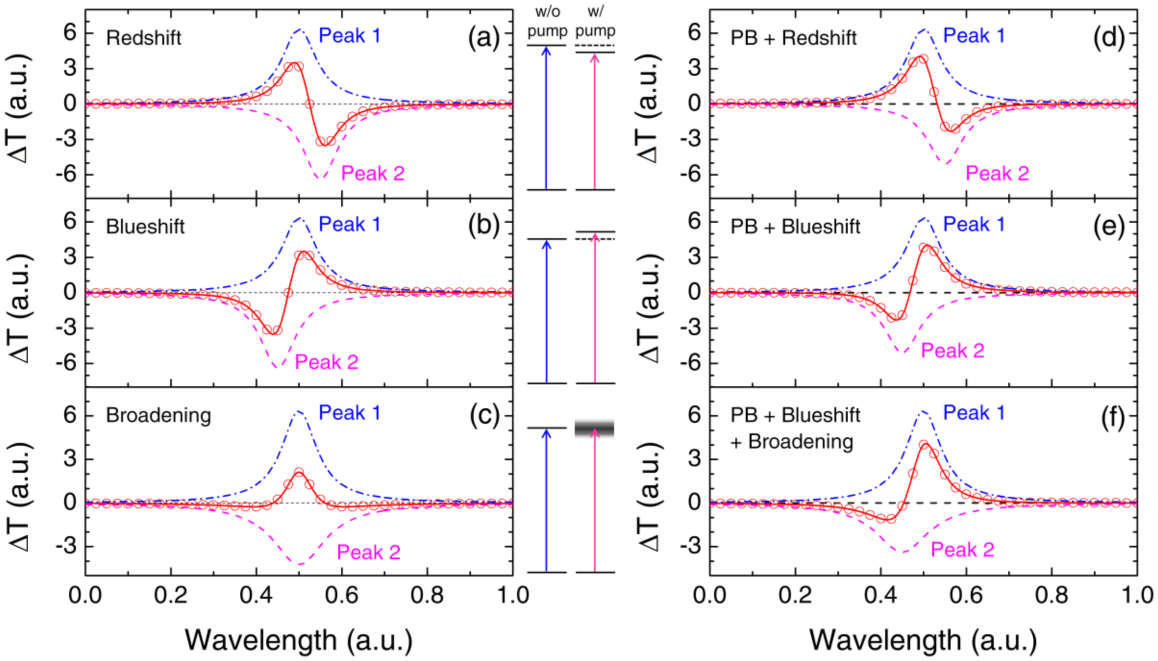

Figure S1 | Simulated transient spectral features of various processes. The simulated transient signal from ground state to exciton state transition for (**a**) redshift, (**b**) blueshift, (**c**) broadening, (**d**) PB + redshift, (**e**) PB + blueshift, and (**f**) PB + blueshift + broadening. All the features can be well-fitted with two Lorentzian peaks.

2. Exciton absorption and transient absorption

Fig S2 shows the two-particle picture of energy-momentum dispersion of excitonic semiconductor. The momentum axis represents the total momentum $K = k_e + k_h$ (*i.e.* center of mass momentum). Similarly, the energy axis also represents the total energy $E = E_e + E_h$. In this picture, the photon is described by a very steep line with gradient $c' = c/n$, where c is speed of light and n is the refractive index of the material. Based on conservation of momentum and energy in absorption process, there are three possible ways of linear absorption process in a direct bandgap semiconductor to occur: (i) direct generation of free hot electron and hole by absorption of photon with energy above bandgap – Fig. S2a; (ii) generation of high momentum exciton by simultaneous absorption of photon ($\hbar\omega > E_g$) and a phonon, similar to indirect bandgap absorption process – Fig. S2b; and (iii) direct generation of exciton by a resonant photon. In our case, the 400 nm (3.1 eV) pump would induce process

(i) and (ii), the 515 nm (2.4 eV) pump would induce process (iii). Relatively broad spectral linewidth (~50 meV) of our laser and absorption broadening of the sample might cause relaxation to the selection rule (*i.e.* 515 nm pump might generates a minor population of free carriers). However, the huge binding energy of PEPI would keep this spectral broadening-induced deviation to be insignificant.

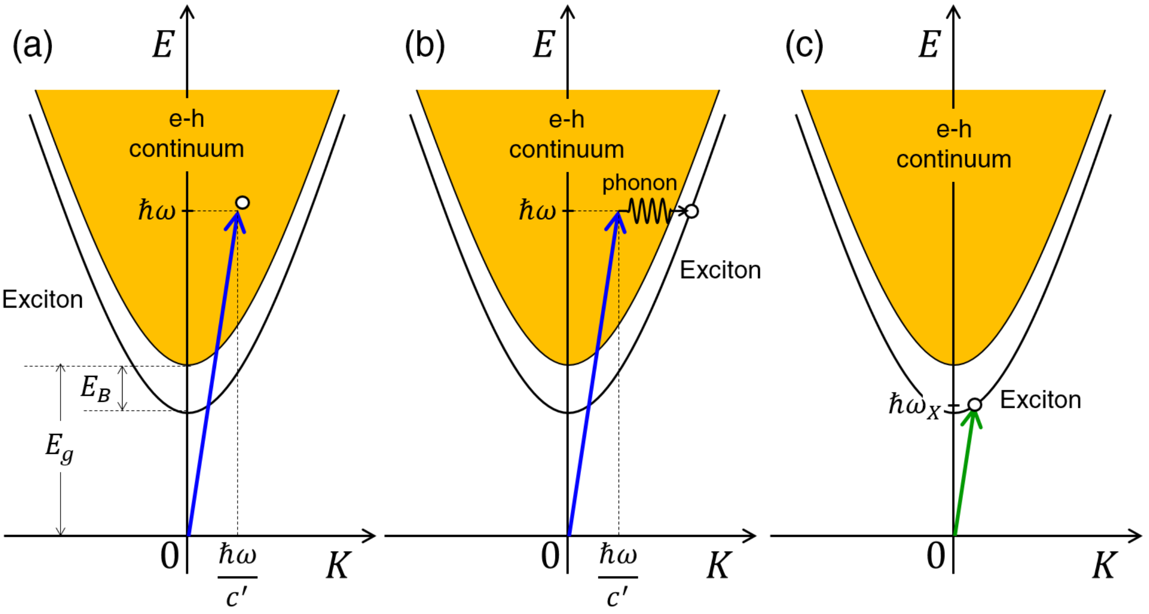

Figure S2 | Two-particle picture of absorption linear process. There are three possible ways of linear absorption process in direct semiconductor bandgap to occur. (a) Generation of free electron and hole by photon with energy $\hbar\omega$ > bandgap E_g. (b) Generation of high momentum exciton by photon with $\hbar\omega > E_g$ and phonon absorption. (c) Direct generation of exciton by resonant photon $\hbar\omega_x$. Here, E_B represents the exciton binding energy.

Fig. S3a shows the comparison of transient spectra between resonant (*i.e.* $\hbar\omega = \hbar\omega_x = 2.4$ eV) and off-resonant ($\hbar\omega = 3.1$ eV) pumping at 5 ps probe delay. Almost identical spectra indicates that the observed post-thermalization dynamics (*i.e.* $t > 2$ ps – Sect. 3) mostly originates from the excitonic state. This observation is consistent with the high binding energy of ≥ 150 meV of our sample. Boltzmann distribution dictates a photoexcited free

carrier to exciton ratio of $\exp(-E_B/k_BT)$ ~ 0.3%, *i.e.* 99.7% of our photoexcited populations are excitons. Fig. S3b shows the rise dynamics of excitonic bleaching signal by 400 nm and 515 nm pump (~200 fs – limit of our temporal resolution). There is no significant difference in the rise-time is observed (< 50 fs), which could come from slight variation of our temporal resolution of different pump wavelength. This signifies an ultrafast hot carrier relaxation and exciton formation (post 400 nm pump excitation) in PEPI beyond our temporal resolution. Hence, it is conclusive that our TA signal would mainly originate from a population of excitons.

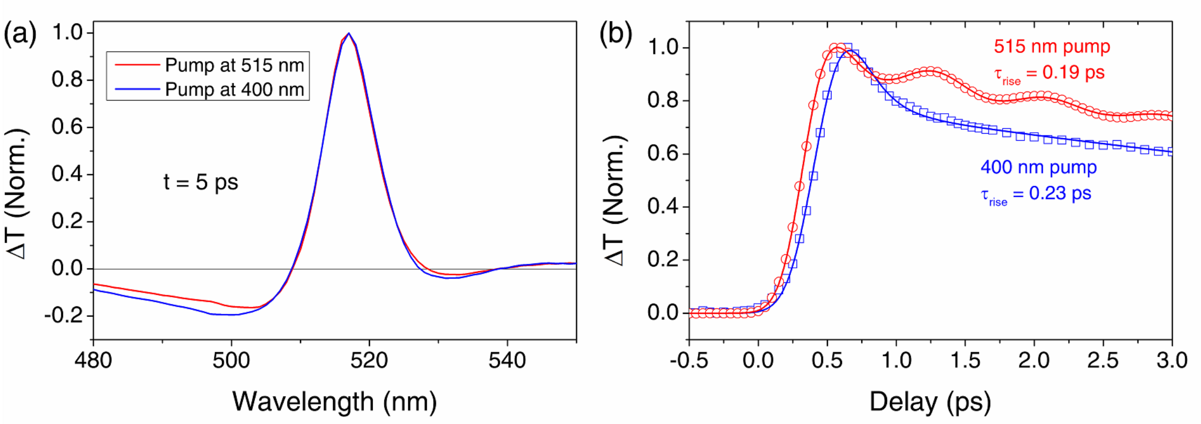

Figure S3 | Transient absorption spectra in PEPI with resonant and non-resonant pumping. (a) Spectral comparison of resonant (515 nm, red) and off-resonant (400 nm, blue) pumping at t = 5 ps. Almost identical spectra implies that the observed dynamics originate mainly from the excitonic state. (b) Rise-time difference between resonant and off resonant pumping.

3. Temporal evolution of TA peaks

Fig. S4(a) shows the decay kinetics of the height of the deconvolved PA1, PB and PA2 peaks from the TA spectra. We performed fitting on the intensity $I(t)$ kinetics of these 3 peaks by multi-exponential decay function:

$$I(t) = \frac{1}{2}\sum_{i=1}^{N} H_i(t)A_i\,exp\left(-\frac{t}{\tau_i}\right) \qquad \text{(S1)}$$

where t is the probe time delay; $H_i(t) = [1 + \mathrm{erf}(-t/r - r/2\tau_i)]$ is the rising function; r = ~0.1 ps is the Gaussian laser pulse width; A_i is the amplitude or pre-exponential function; and τ_i is the decay time. The minimum number of components N to satisfactorily fit the experimental data is 4. From the fitting, we discovered that the kinetics of these 3 peaks can be fitted with four similar lifetimes: $\tau_1 = 0.25 \pm 0.01$ ps; $\tau_2 = 10 \pm 3$ ps; $\tau_3 = 110 \pm 20$ ps; and $\tau_4 = 2000 \pm 600$ ps. The origins of these decays are discussed in the main text. Here, A_1 is assigned to thermalization (discussed later). After thermalization process has completed (i.e. A_1 at $t \gg \tau_1$), these 3 peaks are also observed to share identical dynamics [*i.e.* shown by their identical lifetimes (τ_2, τ_3, and τ_4) and weightage ratio ($A_2 : A_3 : A_4 \approx 1 : 1.4 : 0.47$)]. Such observation implies that these 3 transient peaks originate from a similar exciton reservoir. Hence, any dynamics from this exciton reservoir are identically reflected by the dynamics of those 3 peaks.

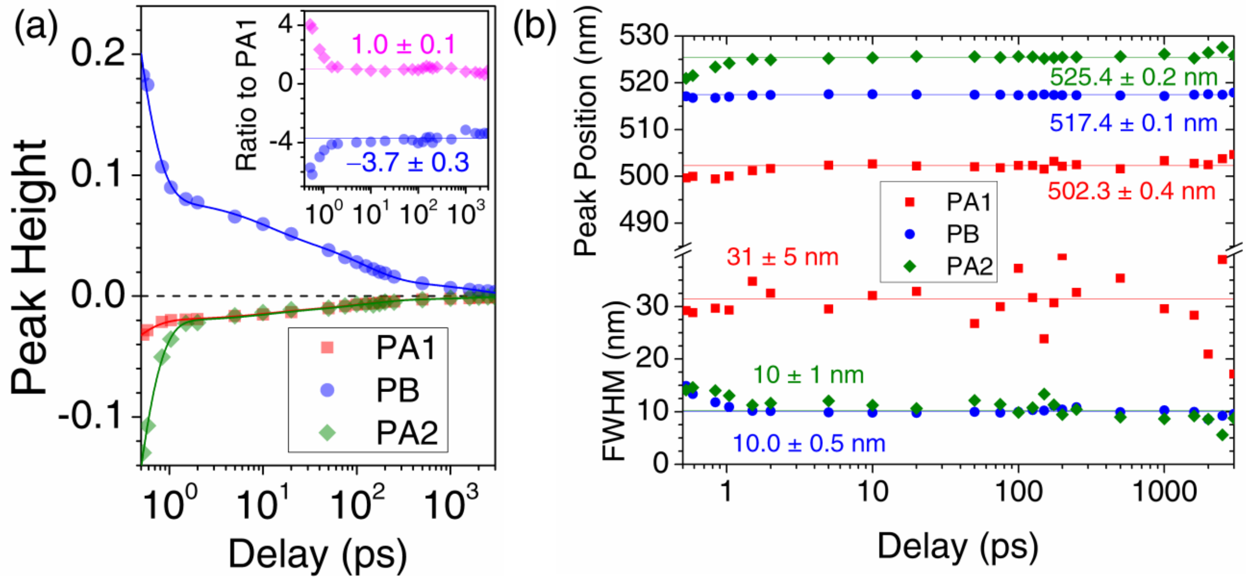

Figure S4 | Peak evolutions of PEPI. (**a**) Kinetics of each Lorentzian peak: PA1 (red square), PB (blue circle) and PA2 (green diamond). Inset: ratios of PB/PA1 and PA2/PA1. (**b**) Peak positions and FWHMs of the

transient 3 Lorentzian PA1, PB and PA2 signals. The peak positions and the FWHMs red-shifted and narrowed, respectively in the first ps of the dynamics. After 1 ps, the peak positions and FWHMs are more or less static.

Consequently, inset of Fig. S4(a) shows constant height ratio of the PB and PA2 peaks to PA1 peak for $t > 2$ ps. Fig. S4(b) shows the constant peak position and peak FWHM for $t > 2$ ps. These observations therefore further support our assignment in the main manuscript that PA1 and PA2 do not originate from carrier induced broadening. If it was, one would expect a recovery of the FWHM and peak position as the population of photoexcited carriers become depleted over time.

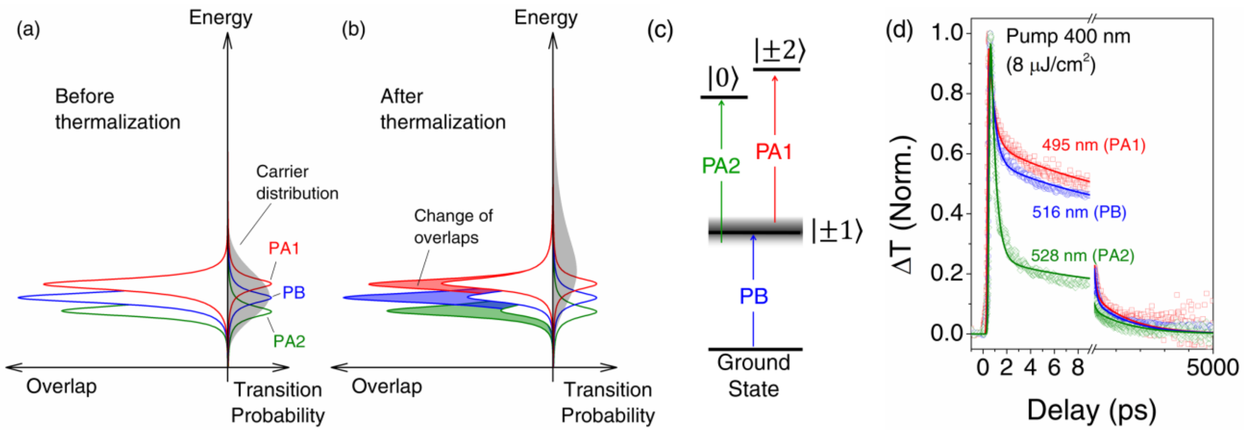

Figure S5 | Illustration of thermalization process of photoexcited exciton in PEPI. (**a**) Before and (**b**) After thermalization. The transient transition probability of PA1 (red), PB (blue) and PA2 (green) peaks overlaps differently with the exciton distribution, resulting in different peak amplitudes. After thermalization, the evolved exciton distribution overlaps differently with the transient probabilities, resulting in different changes of the peak amplitudes. Color filled areas show the change in overlaps before and after thermalization (*i.e.* all the red, blue and green overlap peaks show different decrease after thermalization). This is reflected as different A_1 components. (**c**) Schematic of the transient transition. (**d**) Experimental kinetics at the positions of the 3 transient peaks: PA1 (red), PB (blue) and PA2 (green). The kinetics clearly show different A_1 (fast) components.

The thermalization process assigned to A_1 here is the redistribution or the photoexcited excitons to follow the Bose-Einstein distribution. Such fast lifetime from exciton thermalization is also commonly observed in inorganic QWs.[6] Each of the transient transition peak (*i.e.* PA1, PB and PA2) will have its own spectral transition probability. The amplitude of these transient peaks will be determined by the spectral overlap of the photoexcited exciton distribution (*i.e.* occupation probability) and their transition probability. During thermalization process, the evolving exciton distribution creates ratio changes of its spectral overlap with each of the transition probability of these 3-transient features, resulting in different A_1 components for each of the peak. This process is illustrated in Fig. S5(a) and (b). Different A_1 components are also apparent from the kinetic data extracted at the 3 peaks positions [Fig. S5(d)]. After the thermalization process has completed (t > 2 ps), the exciton distribution becomes more or less static. Hence, the spectral overlap ratio of the exciton distribution and transition probability between these 3 peaks becomes constant. This results in the similar A_2:A_3:A_4 ratio between the 3 peaks. This is also consistent with their constant FWHM and peak positions [Fig. S4(b)].

4. Assignment on the kinetic lifetimes

As a test of our proposed model (involving m_J states) in the main manuscript, fluence dependent study of PEPI thin film are performed. Fig. 2(a) in the Main Text shows the excitation with 400 nm and probing at 517 nm. The kinetics are globally fitted by eq. (S1) across fluences. The fitting yields lifetimes of (τ_1 = 0.43 ± 0.02 ps; τ_2 = 8.5 ± 0.2 ps; τ_3= 90 ± 2 ps; and τ_4 = 1500 ± 30 ps) consistent with our result in Fig S4(a). It has been established that the first lifetime component can be assigned to the exciton thermalization process, with relaxation time constant < 0.5 ps. Since the fraction of excitons undergoing thermalization is

not expected to change with exciton density, it is consistent with the observed fluence-independent A_1 behavior.

Meanwhile, the contribution from A_2 component is observed to increase with fluence, followed by concomitant drop of contribution from A_3 component, which signifies weight transfer from A_3 to A_2 with increasing fluence (Fig. 2(b) in Main Text). Based on this behaviour and its lifetime, we attribute A_3 (~100 ps) process as the monomolecular recombination, consistent with previous reports.[7,8] The A_2 process is attributed to exciton-exciton annihilation (*i.e.* density dependent process), which occurs in a faster timescale (~10 ps). Lastly, A_4, due to its long lifetime and decaying contribution with increasing fluence, is assigned to of trapping/de-trapping process.

5. Phenomenological modelling of TA component contributions

There are several assumptions for the model: (1) A_1 is the fraction of carriers which undergoes thermalization, which is fluence independent. (2) The probability of exciton-exciton (XX) annihilation process (*i.e.* bimolecular – A_2) to occur would be $\propto n^2$ (where n is the exciton population), while the probability of monomolecular process (A_3) to occur would be $\propto n$. Hence, $A_3/A_2 \propto 1/n$. (3) The system has total trap density of n_T^0 with rate of excitons trapping $dn_T(t)/dt$ proportional to the trapping rate k_T and the number of free excitons $n(t)$ as:

$$\frac{dn_T(t)}{dt} = k_T n(t)[n_T^0 - n_T(t)]. \tag{S2}$$

Given the total number of photoexcited excitons n_0, we can straightforwardly solve the differential equation to obtain the fraction of trapped excitons, which is assigned to A_4:

$$A_4(n_0) = x_0 + \frac{n_T^0}{n_0}(1 - e^{-\gamma n_0}). \tag{S3}$$

Here, x_0 is additional offset and γ is a constant, with: $\int_0^\infty n(t)dt \propto n_0$. Since all the components should add up to 1 (or 100%), we can write the following relation:

$$A_1 + A_2(F) + A_3(F) + A_4(F) = 1. \quad \text{(S4)}$$

Here, F is the pump fluence, which is proportional to n_0; A_1 is constant; and $A_4(F)$ is given by eq. S3. This relation could be rearranged:

$$A_2(F) + A_3(F) = 1 - A_1 - A_4(F). \quad \text{(S5)}$$

For A_2 and A_3 components, based on assumption (2), they are related by $A_2 = \beta F A_3$; where β is a constant related to monomolecular and bimolecular coefficient. Substituting this relation to eq. S5, we obtain explicit expression for each component:

$$A_1 = \text{constant} \quad \text{(S5)}$$

$$A_2(F) = \frac{1 - A_1 - A_4(F)}{1 + \beta F}\beta F \quad \text{(S6)}$$

$$A_3(F) = \frac{1 - A_1 - A_4(F)}{1 + \beta F} \quad \text{(S7)}$$

$$A_4(F) = x_0 + \frac{F_T^0}{F}(1 - e^{-\gamma F}). \quad \text{(S8)}$$

Here, $F_T^0 = (\hbar\omega/\alpha)n_T^0$ is the fluence needed to fill the trap; $\hbar\omega$ is the pump photon energy; α ~9×10^4 cm^{-1} is the absorption coefficient; and γ is a constant. This set of equations is then used to simultaneously fit all the components in Fig. 2(b) in the Main Text, with shared parameters. The result shows that all components contributions are well described by eq. (S5)-(S8), which further support our assignment on them. The fitting yields an exciton trap density of $(2.1 \pm 0.3) \times 10^{17}$ cm^{-3}, which is comparable to typical 3D perovskites thin films.[9-11] Additional small fluence independent (x_0 = 2.1% ± 0.7%) offset is also elucidated by the fitting. This offset is assigned to the contributions of the dark excitons ($m_J = 0$) to A_4.

6. Model for transient exciton dynamics in 2D perovskites

The model's temporal component model is adapted based on transient quad-exponential decay function; while the spectral component is the summation of three Lorentzian function based on the observed transient excitonic transition. The transient signal at probe wavelength λ and time delay *t* is given by:

$$\frac{\Delta T}{T}(\lambda,t) = \sum_{i=1}^{3} L_i(\lambda,t) \sum_{j=1}^{4} \frac{C_{ij}}{2}\left[1 + \operatorname{erf}\left(\frac{t}{r} - \frac{r}{2\tau_j}\right)\right] \exp\left(-\frac{t}{\tau_j}\right). \quad \text{(S4)}$$

The parameter *r* refers to the laser time constant; C_{ij} refers to the coefficient; τ_j refers to the decay lifetime; and $L_i(\lambda,t)$ refers to the Lorentzian peak function at a particular time *t*. The dependence of L_i on time is implicit within its center peak position, which oscillates due to coherent exciton-phonon coupling and red shifts during the thermalization process. The function $L_i(\lambda,t)$ can be mathematically described as:

$$L_i(\lambda,t) = \frac{2A_i}{\pi} \frac{\lambda_{Fi}}{4\left[\lambda - \lambda_{C_i} - \lambda_{Ti}(t) + \lambda_O(t)\right]^2 + \lambda_{Fi}^2}. \quad \text{(S5)}$$

Here, A_i is the Lorentzian peak area; λ_{Fi} is the Full Width Half Maximum (FWHM) of the Lorentzian peak; $\lambda_{Ti}(t) = \lambda_{Ti}^0[1 + \operatorname{erf}(t/\tau_i)]/2$ is the peak position shift due to thermalization; $\lambda_0(t) = \lambda_O^0 \cos(2\pi f + \phi) \operatorname{erf}(t/r - r/2\tau_O) \exp(-t/\tau_O)$ is the oscillatory component; λ_O^0 is the oscillation amplitude; f is the oscillation frequency; and τ_0 is the coherence time. The parameter used for the model can be summarized in the table below.

Table S2. Modelling parameters used for phenomenological model fitting.

Parameters	Value	Parameters	Value
r	0.123 ps	C_{11}	1.495
τ_1	0.374 ps	C_{21}	3.346
τ_2	13.1 ps	C_{31}	7.052
τ_3	124 ps	$C_{12} = C_{13} = C_{14}$	0.707

A_1	0.299	$C_{22} = C_{23} = C_{24}$	1.394
A_2	0.502	$C_{32} = C_{33} = C_{34}$	0.482
A_3	0.187	λ_{T1}^0	5.365 nm
λ_{F1}	27.1 nm	$\lambda_{T2}^0 = \lambda_{T3}^0 = \lambda_{T4}^0$	0 nm
λ_{F2}	10.4 nm	λ_O^0	0.4 nm
λ_{F3}	11.8 nm	f	1.15 THz
λ_{C1}	501.3 nm	ϕ	0
λ_{C2}	517.9 nm	τ_O	1.20 ps
λ_{C3}	524.1 nm	$\boldsymbol{R^2}$	**0.9734**

Based on the model, we were able to satisfactorily global-fit the transient data across the whole spectral and temporal window of our measurements, with $R^2 = 0.9734$.

7. Calculation of phonon modes

First-principles calculation of phonon calculation of the compound has been carried out using the Vienna Ab-initio Simulation Package (VASP). The PAW pseudopotentials have been used. The relatively high cutoff energy of 500 eV is for atomic relaxation and subsequently the phonon frequency calculations. The total energies are tested to converge within 1 meV per atom. We simulate the structure in the space group $P12_1/a1$ with a = 32:508 Å, b = 6:131Å, c = 6:185 Å, and β = 93.80°. The result of the phonon frequency and its intensity are tabulated below. The Raman decomposition is:

$$\Gamma = 66A_g + 66B_g + 75A_u + 75B_u.$$

Since the point group is C_{2h}, the Raman active modes are A_g and B_g. The lists of all the correlated Raman frequencies between experiment and first-principle calculation based on VASP, is shown in the table below.

Table S3. Comparison between the correlated Raman frequencies of the calculated phonon modes and the experiment.

Raman mode	Calculation (cm^{-1})	Experiment (cm^{-1})
B_g	38.67	37.3
A_g	39.19	39.8
A_g	53.31	53.7
B_g	55.38	56.6
B_g	96.12	97.4
A_g	96.90	-
A_g	135.82	142
B_g	180.06	165.4
A_g	180.51	-
A_g	252.66	257.3

Regarding the calculations, the frequencies and the modes are computed via VASP using PBE. The non-resonant Raman coefficients was calculated using second-order response as implemented in Quantum ESPRESSO[12] QE setting: DFT calculations carried out within the Quantum ESPRESSO are using the local density approximation (LDA). We use pseudopotentials rel-pz-n-nc.UPF and a kinetic energy cut-off of 60 Ry for electronic wavefunctions in a plane-wave basis set. A Monkhorst-Pack k-point mesh (1 × 3 × 3) is used. The convergence threshold on forces for ionic minimization is 0.38D-4 (a.u.).

Regarding the space group, the (*a, b, c, β*) of Calabrese should contain 2 molecules of $(C_6H_5C_2H_4NH_3)_2PbI_4$ with density of 2.589 g/cm^3. If we stick in the 8 operations SG12, we

will generate 8 copies of $C_6H_5C_2H_4NH_3^+$ (meaning 4 molecules), which is erroneous. This the reason why we had restricted the search to 4 operations SGs which are 5, 8, 9, 10, 11, 13, and 14. All the structures in the different SGs have been relaxed and they were based on lowest total energy criterion that we had applied in the SG14 $P12_1/a1$ setting.

8. Comparison of Raman Spectra

We performed Raman studies using PEPI single-crystal sample and compared it with highly disordered dropcasted PEPI sample– (Fig. S6). The spin-coated PEPI thin-films used in our TA studies were too thin for any appreciable Raman signal. For $\nu < 100$ cm^{-1}, the Raman modes for both samples are well correlated to each other, which implies that they are morphology-independent and originate from intrinsic vibrational modes of 2D perovskites crystal structure. Hence, the main mode of interest ($\nu \sim 40$ cm^{-1}) is universally present.

Meanwhile, morphology-dependent peaks are shown at $\nu > 100$ cm^{-1}. While there is no appreciable Raman feature for single-crystal sample, the dropcasted sample displays several strong peaks with strong asymmetry between Stokes and anti-Stokes peak intensities. We infer that these spectral features at ν > 100 cm-1 could originate from the structural and crystalline disorders in the dropcasted sample. Nevertheless, these morphology-dependent vibrational modes are inactive during our TA measurement and does not affect any of our interpretation.

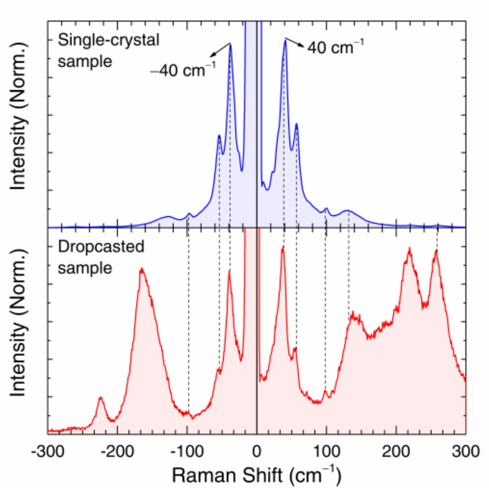

Fig. S6 | Comparison between Raman spectra of single-crystal and dropcasted sample. The dashed line shows the correlated peaks between the two spectra. The labelled modes (~40 cm^{-1}) are the mode of interest.

REFERENCES

[1] G. N. Ostojic, S. Zaric, J. Kono, V. C. Moore, R. H. Hauge & R. E. Smalley, *Phys. Rev. Lett.* **2005**, 94, 097401.

[2] D. Giovanni, W. K. Chong, H. A. Dewi, K. Thirumal, I. Neogi, R. Ramesh, S. Mhaisalkar, N. Mathews & T. C. Sum, *Sci. Adv.* **2016**, 2, 1600477.

[3] V. sharma, S. Aharon, I. Gdor, C. Yang, L. Etgar & S. Ruhman, *J. Mater. Chem. A* **2016**, 4, 3546.

[4] H. Chung, S. I. Jung, H. J. Kim, W. Cha, E. Sim, D. Kim, W.-K. Koh & J. Kim, *Angew. Chem. Int. Ed.* **2017**, 56, 4160.

[5] M. T. Trinh, X. Wu, D. Niesner & X. Y. Zhu, *J. Mater. Chem. A* **2015**, 3, 9285.

[6] T. Amand & X. Marie, *Exciton Spin Dynamics in Semiconductor Quantum Wells*, Springer Berlin Heidelberg, 2008.

[7] K. Abdel-Baki, F. Boitier, H. Diab, G. Lanty, K. Jemli, F. Lédée, D. Garrot, E. Deleporte & J. S. Lauret, *J. Appl. Phys.* **2016**, 119, 064301.

[8] T. Ishihara, J. Takahashi & T. Goto, *Phys. Rev. B* **1990**, 42, 11099.

[9] S. S. Lim, W. K. Chong, A. Solanki, H. A. Dewi, S. Mhaisalkar, N. Mathews & T. C. Sum, *Phys. Chem. Chem. Phys.* **2016**,

[10] T. C. Sum, N. Mathews, G. Xing, S. S. Lim, W. K. Chong, D. Giovanni & H. A. Dewi, *Acc. Chem. Res.* **2016**, 49, 294.

[11] G. Xing, N. Mathews, S. S. Lim, N. Yantara, X. Liu, D. Sabba, M. Grätzel, S. Mhaisalkar & T. C. Sum, *Nat. Mater.* **2014**, 13, 476.

[12] M. Lazzeri & F. Mauri, *Phys. Rev. Lett.* **2003**, 90, 036401.
